# Early detection of ureteropelvic junction obstruction in neonates with prenatal diagnosis of renal pelvis dilatation using ^1^H NMR urinary metabolomics

**DOI:** 10.1038/s41598-022-17664-4

**Published:** 2022-08-04

**Authors:** Aurélien Scalabre, Yohann Clément, Florence Guillière, Sophie Ayciriex, Ségolène Gaillard, Delphine Demède, Aurore Bouty, Pierre Lanteri, Pierre-Yves Mure

**Affiliations:** 1 CHU Nord de Saint-Etienne, Service de chirurgie pédiatrique, Avenue Albert Raimond, 42055 Saint-Etienne, France; 2 Univ Lyon, Université Jean Monnet, Saint-Etienne, SAINBIOSE, Inserm U1059, Avenue Albert Raimond, 42055 Saint-Etienne, France; 3 Univ Lyon, CNRS, Université Claude Bernard Lyon 1, Institut Des Sciences Analytiques, UMR 5280, 5 rue de la Doua, 69100 Villeurbanne, France; 4 Hospices Civils de Lyon, Inserm, EPICIME-CIC 1407, 69677 Bron, France; 5 Laboratoire de Biométrie Et Biologie Evolutive, Equipe Evaluation Et Modélisation Des Effets Thérapeutiques, Univ Lyon, Université Claude Bernard Lyon 1 - UMR-CNRS 5558, 69000 Lyon, France; 6 Hospices Civils de Lyon, Service de chirurgie pédiatrique, Hôpital Femme Mère Enfant, 69677 Bron, France; 7 Univ Lyon, Université Claude Bernard Lyon 1, Inserm UMR 1208, 18 Avenue du Doyen Jean Lépine, 69500 Bron, France

**Keywords:** Nephrology, Metabolomics

## Abstract

Renal pelvis dilatation (RPD) is diagnosed in utero on prenatal ultrasonography (US) and can resolve spontaneously. However, isolated RPD can also reflect ureteropelvic junction obstruction (UPJO), which requires surgical treatment to prevent progressive renal deterioration. The diagnosis of UPJO can only be confirmed after birth with repeat US and renal isotope studies. ^1^H Nuclear Magnetic Resonance spectroscopy (NMR) was performed on urine of newborns with prenatally diagnosed unilateral RPD and healthy controls to identify specific urinary biomarkers for UPJO. The original combination of EigenMS normalization and sparse partial-least-squares discriminant analysis improved selectivity and sensitivity. In total, 140 urine samples from newborns were processed and 100 metabolites were identified. Correlation network identified discriminant metabolites in lower concentrations in UPJO patients. Two main metabolic pathways appeared to be impaired in patients with UPJO i.e. amino acid and betaine metabolism. In this prospective study, metabolic profiling of urine samples by NMR clearly distinguishes patients who required surgery for UPJO from patients with transient dilatations and controls. This study will pave the way for the use of metabolomics for the diagnosis of prenatal hydronephrosis in clinical routine.

## Introduction

Renal Pelvis Dilatation (RPD), sometimes referred as hydronephrosis, is among the most common congenital anomalies diagnosed in 0.2–% of all pregnancies^1,2^. The dilatation is often transient or physiological, spontaneously regressing with no clinical significance^1,3^ in 50–70% of cases. However, isolated RPD can be the first sign of significant urinary flow impairment related to ureteropelvic junction obstruction (UPJO). This diagnosis can only be assessed after birth on repeat ultrasonography (US) and renal isotope studies, with significant consequences regarding childhood radiation, parental anxiety, and use of health care resources^1,3^. Surgical treatment is required to prevent urinary tract infections (UTI), stone formation, and progressive renal function deterioration^4^. Identifying specific urinary metabolites in neonates could help differentiate UPJO from transient dilatation (TD) at an early stage and therefore, prompt surgery to prevent loss of renal function. Identifying biomarkers with this predictive value has been a large field of research for many years. Early fetal urinary tract obstruction is a complex situation resulting in alteration of glomerular, hemodynamic, and tubular functions caused by the interaction of a variety of vasoactive factors and cytokines that are activated in response to obstruction^5–7^. It is associated with an increased urinary concentration of tubular mesenchymal markers *i.e.*, transforming growth factor beta 1^8,9^, β-catenin, and vimentin, an increased production of epidermal growth factor^10–13^ and cell adhesion molecules, kidney injury molecule-1, and neutrophil gelatinase-associated lipocalin^14^, and a decrease in epithelial markers (aquaporin-2 and E-cadherin)^15^. A recent proteomics study showed that a panel of five proteins including prostaglandin-reductase-1, ficolin-2, nicotinate-nucleotide pyrophosphorylase [carboxylating], immunoglobulin superfamily containing leucine-rich-repeat-protein and vascular cell adhesion molecule-1 were present at higher levels in the UPJO samples compared to controls^16^. However, previous studies investigating potential biomarkers for UPJO were conducted on small population samples, and clear indications for surgery were often lacking. None of these studied molecules is currently used in clinical practice to differentiate transient RPD from UPJO.

Metabolomics is an emerging ‘omics’ field devoted to investigate global metabolic profiles in biological samples. It offers a comprehensive view and a dynamic snapshot of the metabolic status of a biological system^17^. This approach involved advanced analytical techniques *i.e.* liquid chromatography or gas chromatography coupled to high-resolution mass spectrometry or Nuclear Magnetic Resonance (NMR) spectroscopy. NMR spectroscopy is widely used in metabolomics since it is a non-destructive technique, easy to set up with no tedious sample treatment. It offers high reproducibility and easy quantification. Besides these advantages, NMR is particularly well suited for the detection and characterization of compounds with low molecular weight such as amino acids, sugars, polar compounds, lipids.

In this study, we evaluated and used proton NMR (^1^H-NMR) spectroscopy combined with EigenMS normalization and sparse methods to identify specific urinary biomarkers for early detection of UPJO in neonates and by consequence for the characterization of metabolic pathway alterations. Sparse partial-least-squares discriminant analysis was applied and provided a robust classification. High sensitivity and specificity models is necessary for the discrimination between urine samples from controls, transient dilatations and patients who required surgery for UPJO. Metabolites responsible for these differences were identified. Analysis of urinary metabolome appeared to be a valuable tool for predicting surgical UPJO repair in newborns with prenatally diagnosed unilateral RPD.

## Material and methods

### Chemicals

Deuterium oxide (D_2_O), 3-(trimethylsilyl)propionic-2,2,3,3-*d*_*4*_ acid sodium salt (TSP), phosphate buffer and sodium azide were purchased from Sigma-Aldrich (St Quentin Fallavier, France). All reagents were of analytical grade. Deionised and distilled water were used throughout.

### Subjects

Children with prenatally diagnosed unilateral RPD (n = 50) (28 left sided and 22 right sided) were prospectively included from February 2011 to January 2015 in the University hospitals of Lyon. Other inclusion criteria were age < 4 months, intrarenal antero-posterior diameter (APD) ≥ 10 mm on the first postnatal ultrasound (US), ipsilateral ureter diameter of less than 6 mm, and normal bladder (Table S1a,b).

Newborns (n = 90) consulting at our institution for a benign condition (42 inguinal hernia, 27 abdominal discomforts, 11 suspicion of pyloric stenosis, 5 undescended testis, 3 umbilical granuloma and 2 umbilical hernia) were included as controls during the same period (Table S1a,b). Patients with prematurity (gestational age of less than 38 weeks), history of metabolic, nephrological or urological diseases were not eligible as controls.

The values reported in the Table S1a were established at the time of collection.

### Ethical statement

The study was approved by a French ethical committee (CPP, Sud Est II, Oct 6, 2010). All methods were carried out in accordance with relevant guidelines and regulations. The study was approved by the regional ethical committee and informed written consent was obtained from all parents.

### Urine sample collection

Urine sample was collected non-invasively from each patient and control during a medical consultation within 120 days from birth. The genitals were cleaned thoroughly with warm water and soap and dried with sterile gauze. A sterile bag was placed on the genital area until micturition. Samples were stored in sterile tubes at − 80 °C before NMR data acquisition.

Patients underwent urinary tract imaging according to a systematic protocol including serial US and isotope studies. Clinical and imaging data were recorded prospectively. Indications for surgery were increasing APD greater than 20%, ipsilateral split renal function of less than 40%, decrease of relative renal function more than 10% and recurrent febrile UTIs. Consents were obtained from parents, before any study procedure was performed.

### Urine sample preparation

Samples collected during the medical consultation within 120 days from birth were analyzed in randomized order. An additional series of quality control (QC) urine samples (n = 15) was included in the analysis to evaluate the reproducibility of the NMR data acquisition. The QC samples were obtained by pooling a fraction of the first 12 urine samples. QC samples were regularly analyzed throughout the run. Urine samples were thawed at room temperature and centrifuged at 12,000 g at 4 °C for 5 min. 540 µL of the supernatants were mixed with 60 µL of phosphate buffer in D_2_O (1.5 M, pH = 7.4) containing 1% of 3-(trimethylsilyl)propionic-2,2,3,3-*d*_4_ acid sodium salt, 98 atom %D (TSP) as internal standard (final TSP concentration = 0.77 mM) and 3 mM sodium azide to prevent microbial contamination and then transferred into 5 mm NMR sample tubes. Samples were kept at 4 °C before analysis.

### ^1^H-NMR spectroscopy of urine samples

All NMR experiments were performed on a Bruker Avance III spectrometer operating at 600.55 MHz (proton resonance frequency) equipped with a cryo-probe, and high-throughput sample changer that maintained the samples temperature at 4 °C until actual NMR acquisition. All samples were analyzed in one batch for 14 days. For each session, Automatic 3D shimming was performed once on a QC sample. Prior to NMR data acquisition, automatic tuning and matching, frequency locking on D_2_O and 1D automatic gradient shimming was performed on each sample. The temperature was regulated at 300 K throughout the NMR experiments. Standard ^1^H 1D NMR NOESY pulse sequence with water-suppression using Presaturation pulse was applied on each sample to obtain corresponding metabolic profiles. 200 and 56 transient free induction decays (FID) were collected for each experiment into 48,074 data points over a spectral width of 20 ppm. The acquisition time was set to 2 s with a relaxation delay of 4 s. The NOESY mixing time was set to 100 ms. The 90° pulse length was automatically calibrated for each sample at around 14.24 µs. In addition, 2D NMR experiments (^1^H-^13^C HSQC, ^1^H-^1^H TOCSY and J-Resolved) were recorded on a subset of samples to achieve structural assignment of the metabolic signals. Metabolites were assigned from the mean NOESY and 2D spectra using data available from the literature^18^, international reference database (Human Metabolic Database, HMDB)^19^ or proprietary databases (Chenomx NMR Suite 8.0, Chenomx Inc., Edmonton, Canada, https://www.chenomx.com).

### NMR data processing

Data were imported into R (v4.0.2, https://www.r-project.org) for data processing using PepsNMR package (v1.10.1, https://github.com/ManonMartin/PepsNMR^20^. All FIDs were multiplied by an exponential function corresponding to a 0.3 Hz line-broadening factor, prior to Fourier transformation. ^1^H-NMR spectra were phased, baseline corrected and referenced to the TSP signal at − 0.016 ppm. The spectra were realigned based on a reference spectrum with a Semi-Parametric Time Warping technique. Residual water signal (4.6–5.2 ppm) was excluded. Spectra were divided into 0.01 ppm-wide buckets over the chemical shift range [0; 10 ppm]. The data matrix resulting from processing has 140 samples and a dimension of 1000 variables (buckets).

### Multivariate statistical analysis

Data were analyzed using function on MixOmics package (v6.16.3, http://mixomics.org) (R package) for statistical analyses. Principal Component Analysis (PCA)^21^ and Sparse PCA (SPCA)^22^ were performed an all samples. NMR data were centered. Stability and reproducibility of NMR experiments were monitored by QC samples.

For normalization, EigenMS package was used. Models were calculated using Sparse Partial Least Square Discriminant Analysis (SPLS-DA)^23,24^ for variable selection. Statistical significance of models was assessed using confusion matrix and by permuting the validation set. External validation was performed by splitting the initial dataset into training and test data. Significance of NMR variables for the effect under study was calculated by multiple testing correction of the p-values^25^ (Benjamini–Hochberg False Discovery Rate). Correlation network was performed with R package “qgraph” (v 1.9)^26^.

## Results and discussion

### Patients and clinical assessments

After a median follow-up of 26 months (mean 26.7, range 4.2–53.5, IQR 14.6–36.8, standard deviation 13.5), 26 patients (52%) required a surgical repair for UPJO (dismembered pyeloplasty) at a median age of 4.7 months (mean 6.9, range 1.7–40.5, IQR 3.4–6.2, standard deviation 8.1), constituting the UPJO group. Indications for surgery were increasing APD greater than 20% in 22 (85%) cases, ipsilateral split renal function of less than 40% in 12 (46%), and recurrent febrile UTIs in 4 (15%). Eight patients (31%) had increasing APD plus alteration of split renal function, 3 patients (12%) had increasing APD plus febrile UTIs and 2 patients had increasing APD, alteration of split renal function and febrile UTIs.

24 patients did not require surgery after a median follow-up of 21.6 months (mean 26.7, range 4.2–53.5, IQR 14.6–36.8, standard deviation 12.8). RPD resolved spontaneously in 14 (54%) cases after a median of 16.7 months (mean 18.3, range 0.4–23.6, IQR 7.5–24.0, standard deviation 13.4), constituting the transient RPD group.

### Urine ^1^H NMR metabolomics

Urine samples within 120 days post birth, from newborns with prenatally diagnosed RPD (n = 50), confirmed on ultrasound scan performed 4 to 10 days after birth, and healthy newborn controls (n = 90) were collected. ^1^H NMR spectroscopy was used to perform metabolic profiling of the 140 newborns urine samples. We identified 100 metabolites from the recorded NMR data set (Table S2). Multivariate data analysis was applied to discriminate between TD patients and newborns that need surgery for UPJO.

### Multivariate data analysis of urine ^1^H NMR Metabolomics data of TD, UPJO and control newborns

Metabolomics data can suffer from the effects of various systematic biases (physical or biological like urine concentration). Usual normalization methods (mean, median, cubic spline, probabilistic quotient normalization) were tested but no separation was observed between groups using unsupervised methods such as PCA. However, the superposition of the non-normalized average spectra control versus UPJO and UPJO *versus* TD (see Supplementary Fig. S1A-B) exhibited differences on several buckets. It was therefore chosen to normalize the spectra in a supervised manner. To eliminate bias and to separate the groups without any a priori hypothesis, a recent normalization method called EigenMS^27,28^ was applied. The EigenMS normalization was performed in four steps, (i) evaluation of group effects with an ANOVA model, (ii) singular value decomposition of the residuals matrix for the determination of bias trends in the data set, (iii) permutation test for the estimation of bias trends and (iv) elimination of bias. This method proved to be useful on LC–MS and GC–MS data and has been applied for the first time on our NMR data set.

**Figure 1:**
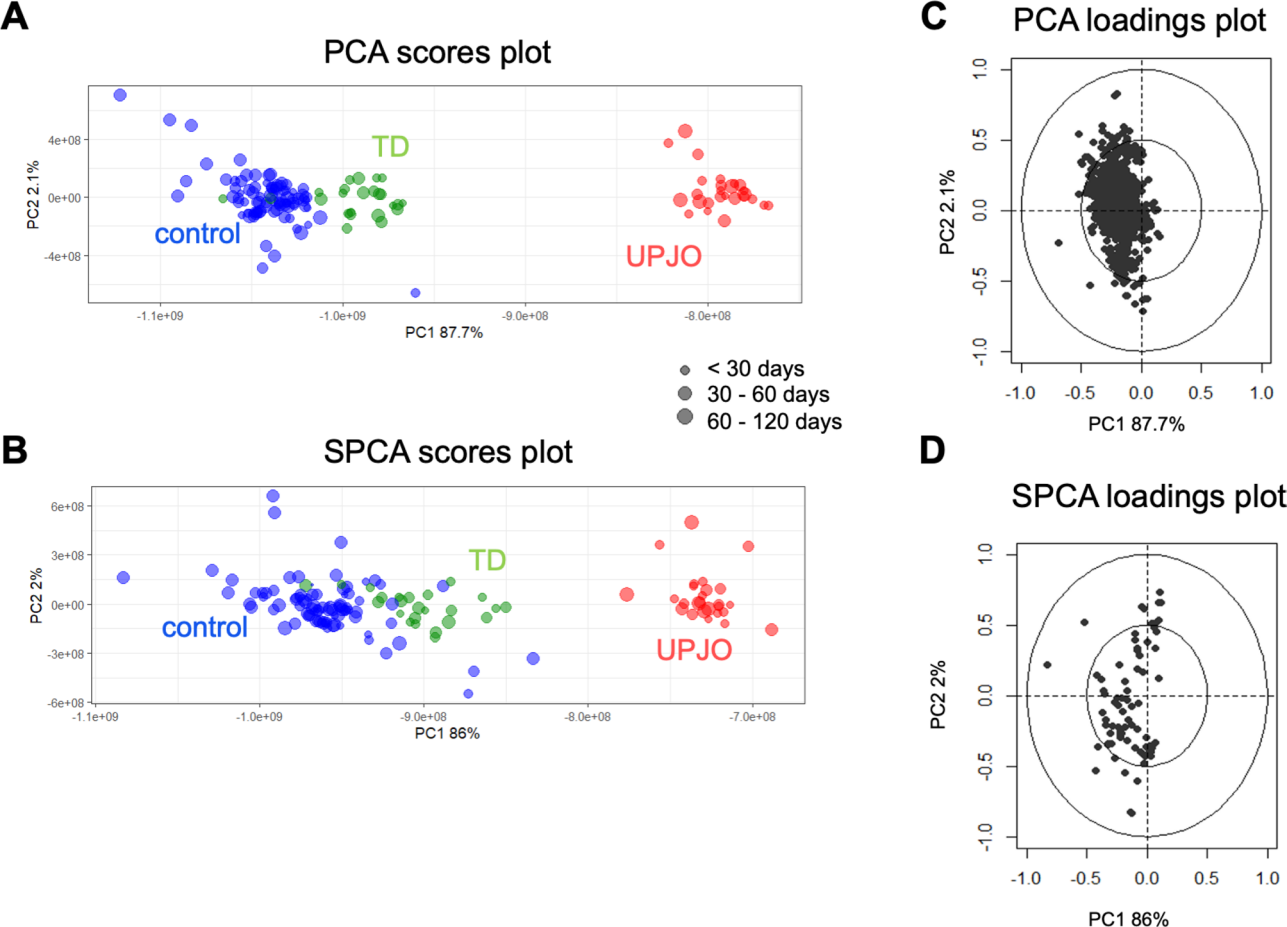
PCA and SPCA analysis of metabolites in urine after ^1^H NMR analysis from TD and UPJO patients. (**A**) PCA scores with 87% of the variance explained on the first component and 2.1% on the second component. (**B**) SPCA scores with 86% of the variance explained on the first component and 2.2% on the second component. Fifty variables are selected on each component. SPCA allows the separation between groups. The different groups of samples are represented by colors: control (blue), TD (green), UPJO (red). The size of the dots varies according to the age of the patient in days (10 to 115). (**C**) Loadings plot from PCA with all variables and (**D**) Loadings plot from SPCA with 50 variables on each principal component.

**Figure 2:**
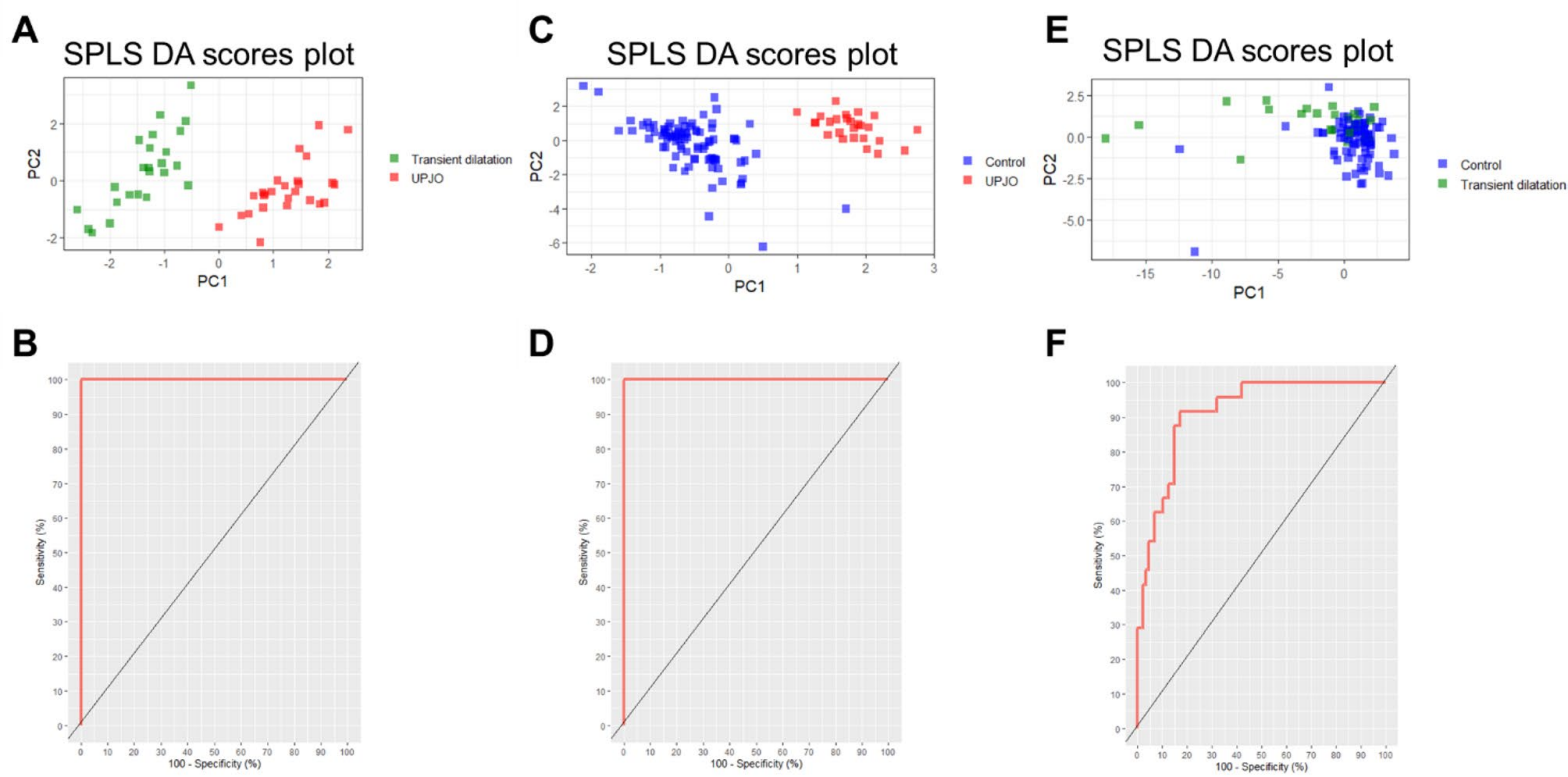
SPLS-DA analysis of UPJO *vs* TD, UPJO *vs* controls and TD *vs* controls. (**A**) SPLS-DA scores plot PC1 (15%) vs PC2 (4%) with a good separation between UPJO (red) vs TD (green) ; (**C**) SPLS-DA scores plot PC1 (14%) vs PC2 (5%) with a good separation between UPJO (red) vs controls (blue); (**E**) SPLS-DA scores plot PC1 (25%) vs PC2 (6%) with a light separation between TD (green *vs* controls blue). For each model classification, a receiver operating characteristic (ROC) curve is shown (**B**,**D**,**F**).

PCA (Fig. 1A,C) and SPCA (Fig. 1B,D) were applied to explore the NMR dataset. The difference between these two descriptive methods is the number of variables on each component, *i.e.* all variables for the principal component of PCA and 50 variables for the principal component of SPCA. TD, controls and UPJO groups were clearly separated. Consequently, specific urinary metabolic profiles could be identified for each group. Selection of variables on each factorial axis of the SPCA allowed discrimination of the differences between groups without loss of information. The influence of age on the urinary metabolome of newborns^29^ was demonstrated in a previous study. However, in the present dataset, discrimination between groups was independent from age at the time of urine sampling. Preliminary SPCA (see Supplementary Fig. S2) was also performed and confirmed that height and weight of newborns did not significantly influence the dataset. The metabolic profiles of UPJO *vs* TD, UPJO *vs* controls, and TD *vs* controls (Fig. 2A–F) were compared with SPLS-DA.

SPLS-DA procedure introduces penalties (l1) on the loadings weights to select variables. A soft-thresholding method was applied^23,24,30^. The number of components for each model and methods was optimized using cross-validation (k-fold cross-validation). For SPLS-DA, overall error rate and Balanced Error Rate (BER)^30^ measured performance of classification to compare the models constructed with different penalties (See Supplementary Fig. S3A-C). The optimal number of components was obtained according to the best performance on the overall error rate or BER^30^. The Receiver Operating Characteristic (ROC) curves and Area Under the Curve (AUC) were used as an additional method of graphical model evaluation. The SPLS-DA models proved significant differences between groups (UPJO *versus* TD and UPJO *versus* controls) as shown by the scores plot on Fig. 2A,C. Statistical significance of models was assessed using confusion matrix. The selected model for comparison of UPJO and TD had two components with respectively 9 and 6 selected variables (Fig. S2). 18 samples (n = 9 per group) were extracted to serve as test data and the predicted group was classified correctly on each permutation (50 permutations), demonstrating a sensitivity and specificity of 100%. The calculated model allows identifying significant differences in urinary metabolic profiles of newborns from the UPJO group who needed surgery within two years after sampling, and newborns with TD. Discriminant variables and corresponding metabolites were annotated in Table 1A. It is important to note that some variables can correspond to several metabolites, as NMR signals from these metabolites have overlapping peaks. Consequently, we reported all metabolites potentially associated with discriminant variables: acetate, ascorbate, betaine, creatine, dimethylglycine, threitol, glucoronate, alanine, arginine, lysine, threonine, *N,N*-dimethylaniline, ornithine, taurine, and trimethylamine N-oxide (TMAO).

**Table 1:** Metabolites identified as key molecules significantly correlated with (A) UPJO vs TD and (B) UPJO vs controls.

ppm	Loadings	Components	Annotation	HMDB	p value	p value adjusted
**(A)**						
3.27	− 0.88	PC 1	TMAO Arginine	00925 00517	4.53E−08	4.29E−05
3.70	− 0.27	PC 1	Diméthylglycine Lysine	00765 00182	4.21E−06	1.53E−03
3.77	− 0.24	PC 1	Alanine Threonine Threitol Ornithine Glucoronate	00161 00943 04136 00214 00625	5.00E−06	1.53E−03
1.82	− 0.19	PC 1	Acetate Arginine Lysine	00042 00517 00182	6.99E−06	1.53E−03
3.69	− 0.16	PC 1	Threonine Threitol Glucoronate	00765 04136 00625	8.13E−06	1.53E−03
3.78	− 0.13	PC 1	Alanine Threitol	00161 04136	1.01E−05	1.53E−03
4.01	− 0.08	PC 1	Ascorbate Glucoronate	00044 00625	1.30E−05	1.53E−03
4.28	− 0.08	PC 1	Threonine	00943	1.32E−05	1.53E−03
2.83	− 0.07	PC 1	*N*,*N*-dimethylaniline	01020	1.45E−05	1.53E−03
3.26	0.95	PC 2	Betaine Taurine	00043 00251	6.73E−03	1.32E−02
3.04	0.22	PC 2	Creatine Lysine Ornithine	00064 00182 00214	2.39E−02	3.58E−02
1.47	0.15	PC 2	Alanine Threonine Lysine	00161 00943 00182	4.92E−03	1.08E−02
3.94	0.14	PC 2	Creatine	00,064	1.83E−02	2.87E−02
3.92	0.1	PC 2	Betaine	00,043	1.52E−02	2.45E−02
7.33	0.05	PC 2	*N*,*N*-dimethylaniline	01,020	1.95E−02	3.03E−02
**(B)**						
3.27	− 0.95	PC 1	TMAO Arginine	00925 00517	2.30E−18	2.18E−15
3.05	− 0.26	PC 1	Creatinine	00562	4.25E−09	2.01E−06
2.51	− 0.08	PC 1	Acetylcarnitine Octanoylcarnitine	00201 00791	1.56E−07	3.22E−05
2.72	− 0.08	PC 1	Dimethylamine	00087	1.63E−07	3.22E−05
6.77	− 0.07	PC 1	Not identified		1.88E−07	3.22E−05
4.07	− 0.06	PC 1	Creatinine	00562	2.35E−07	3.22E−05
2.83	− 0.06	PC 1	*N*,*N*-dimethyaniline	01020	2.38E−07	3.22E−05
6.76	− 0.03	PC 1	Not identified		4.06E−07	4.81E−05
3.04	− 0.90	PC 2	Creatine Lysine Ornithine	00064 00182 00214	4.25E−09	2.01E−06
3.94	− 0.50	PC 2	Creatine	00064	4.27E−02	7.37E−02
3.77	− 0.10	PC 2	Alanine Threonine Threitol Ornithine glucoronate	00161 00943 04136 00214	8.92E−06	4.33E−04
3.26	− 0.95	PC 3	Betaine Taurine	00043 00251	1.75E−05	5.66E−04
2.53	2.53	PC 3	β-Alanine Citrate	00056 00094	3.20E−02	5.82E−02
3.55	− 0.10	PC 3	Glycine	00123	8.04E−01	8.29E−01
3.45	− 0.05	PC 3	Taurine	00251	5.12E−02	8.57E−02

Loadings correspond to the weight of the variables on SPLS-DA components. NMR shifts may correspond to several metabolites (annotation). HMDB column indicate the reference in the HMDB database of each annotated NMR shifts. A parametric (p-value) and non-parametric (p-value adjusted) test were applied to verify significance for NMR shifts.

### Urine metabolic correlation between UPJO and TD

**Figure 3:**
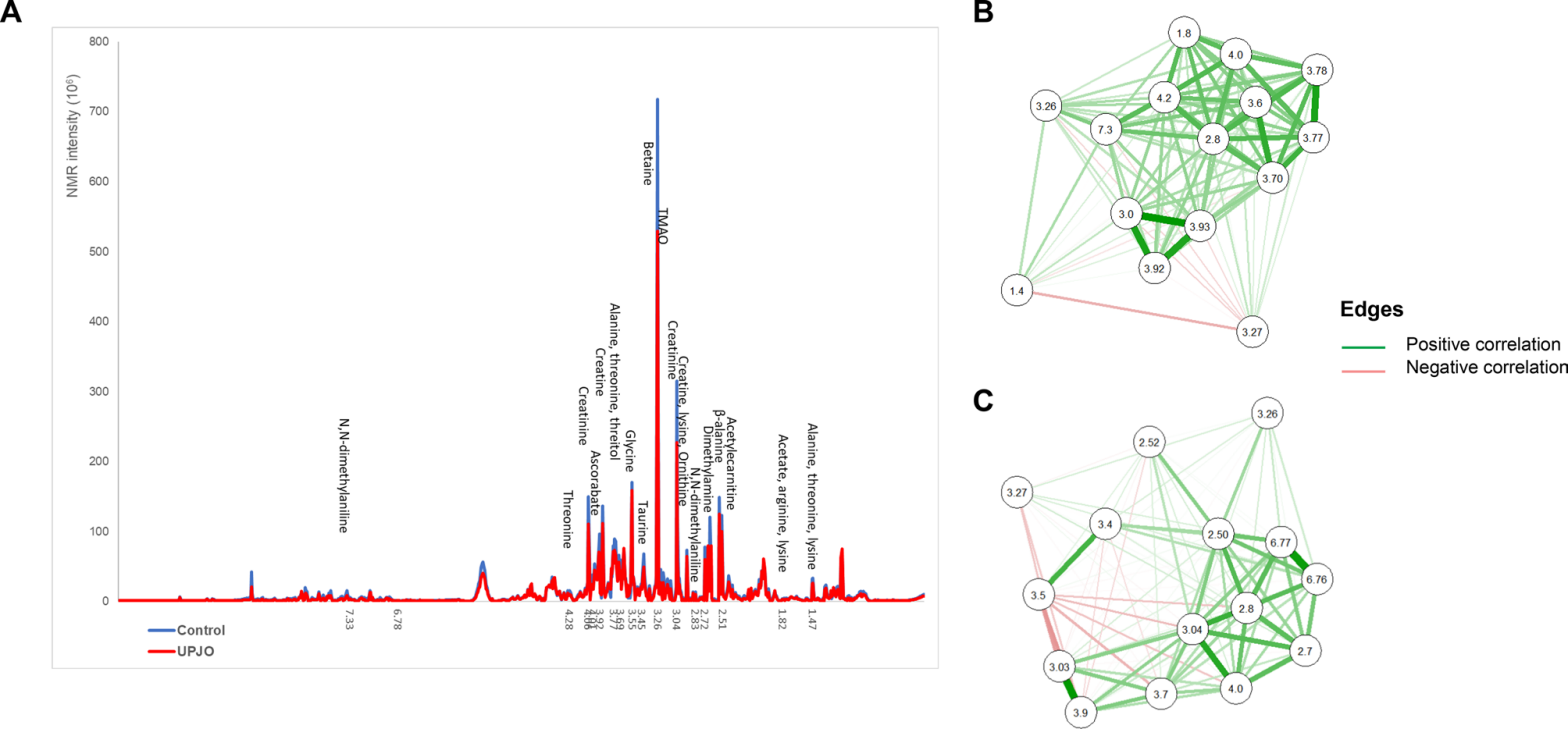
(**A**) Average of NMR spectra from UPJO (red) and control (blue) and bucket’s annotation from SPLS-DA. (**B**)–(**C**) Correlation network of the variables from NMR. (**B**) UPJO *versus* TD (C) UPJO versus controls. The color indicate link between variables—red for anti-correlation and green for correlation. Thickness of lines demonstrates the intensity of the correlation. Correlation network was performed using qgraph R package.

Figure 3A displays the superposition of mean NMR spectra for UPJO and TD. Annotated metabolites with significant BH tests were in lower concentration in the UPJO group compared to the TD group. Correlation networks were constructed to represent the links between variables selected by SPLS-DA analysis (Fig. 3B–C). Correlations between significant variables of the model were calculated. A correlation matrix can be represented as a network in which each variable is a node and each correlation an edge. By varying the width and color (correlated or anti-correlated) of the edges according to the magnitude of the correlation, the structure of the correlation matrix can be visualized. This representation allows better visualization of the correlations between discriminant variables, and thus the links between metabolites found to be in lower concentration in urine samples from the UPJO group. Using HMDB, betaine metabolism as well as glycine and serine metabolism were identified as the two main metabolic pathways modified in patients with UPJO.

Another SPLS-DA model was calculated to discriminate urinary metabolic profile of UPJO from controls. This model had 3 components with respectively 8, 3 and 4 selected variables (see Supplementary Fig. S2). Regarding the confusion matrix, 24 controls and 11 patients with UPJO were extracted to serve as test data, with 50 permutations. The predicted group was classified correctly on each permutation, with only 1 control considered as outlier for one permutation. The model highlighted significant differences between urinary metabolic profiles of newborns with UPJO and controls. Metabolites associated with the discriminant variables are betaine, creatine, threitol, glucoronate, alanine, arginine, lysine, threonine, *N,N*-dimethylaniline, ornithine, taurine, and TMAO (Table 1B). They were identified as key metabolites in our two models, with lower urinary concentrations in the UPJO group.

Our results show that within 120 days from birth, newborns with prenatally diagnosed with unilateral RPD corresponding to UPJO had a specific urinary metabolic profile that can be differentiated from urinary metabolic profile from newborns with TD and controls.

In contrast, the urinary metabolic profiles of controls and newborns with TD were not significantly different. Indeed, the corresponding SPLS-DA model, with 3 components on which respectively 50, 6 and 3 variables were selected (see Supplementary Fig. S2), showed no clear separation between groups. 44 samples (33 controls and 11 with TD) were extracted for validation. Samples from the TD group were not correctly classified on any permutation (50 permutations). Thus, newborns with TD had urinary metabolic profiles that could not be distinguished from profiles of controls. This is consistent with the fact that prenatally diagnosed RPD of these newborns did not correspond to significant urinary flow impairment and, as such, did not need surgery during follow-up.

## Conclusion

Using ^1^H-NMR spectroscopy and multivariate data analysis, we identified a urinary metabolic profile specific to UPJO and therefore predictive of the need for surgery. Newborns with prenatally diagnosed RPD whose urinary profile corresponds to UPJO could be operated earlier, thus limiting the deterioration of the renal function and the risk of urinary tract infections. Metabolomics analysis also allows early identification of newborns whose RPD is likely to be transient and can therefore be treated conservatively. In consequence, the number of imaging studies could be reduced during follow-up, avoiding unnecessary irradiation.

Metabolomics has the advantage of being a top-down approach, without a priori. It is therefore a useful tool for biomarker discovery and identification of specific metabolic profiles. We used EigenMS normalization to remove potential bias from the metabolomics data^27^ and SPLS-DA, which is a performant variable selection tool for highly dimensional data sets^31^. In addition, our strategy revealed correlations between the different variables, allowing undoubtedly identification of a specific urinary metabolic profile associated with UPJO, with potential direct benefits for newborns with prenatally diagnosed RPD.

Further investigations using high-resolution mass spectrometry can be considered to validate the discriminant metabolites and to identify others that could not be detected by ^1^H-NMR. A proteomics study using a comparable study design identified 51 distinct urinary proteins that allowed discrimination between healthy newborns, newborns with UPJO who required surgery and newborns with transient RPD with 98% sensitivity and specificity^32^. Combining proteomics and metabolomics could enable precise identification of metabolites and proteins involved in the pathophysiology of UPJO. Recent metabolomics studies employing HILIC LC–MS or GC–MS revealed metabolic perturbations on serum of UPJO infants requiring surgery compared to a conservative group^33,34^. Decrease in amino acids concentrations and a product of lipid peroxidation biomarker was highlighted in serum of patients with UPJO ^33,34^.

To our knowledge, our study represents the first report of the use of metabolomics by NMR spectroscopy to discriminate between TD patients and newborns that need surgery for UPJO. We believe that this preliminary work will pave the way for the use of metabolomics for the diagnosis of prenatal hydronephrosis in clinical routine.

## Supplementary Information

Supplementary Information.

## Data Availability

The datasets generated during and analyzed during the current study are available from the corresponding author on reasonable request.
