# Supplementary material for: Early detection of ureteropelvic junction obstruction in neonates with prenatal diagnosis of renal pelvis dilatation using 1H NMR urinary metabolomics
Source: Sci Rep. 2022 Aug 4;12:13406. doi: 10.1038/s41598-022-17664-4 (PMC9352869; doi:10.1038/s41598-022-17664-4)
Supplement: Supplementary file 1 — Supplementary Information. [file 41598_2022_17664_MOESM1_ESM.docx]

**Supporting information**

**Fig. S1: Superposed of non-normalized average ^1^H NMR spectra of urine samples from control *versus* UPJO (A) UPJO and TD (B).**

**Fig. S2: SPCA analysis of normalized metabolites in urine after ^1^H NMR analysis.** SPCA scores with 86 % of the variance explained on the first component and 2 % on the second component; 50 variables are retained on each component; SPCA allows the separation between the different groups; the size of the dots vary according to the height (A) and the weight (B).

**Fig. S3: Evolution of Error balance rate according to the number of variables selected by components.** A diamond indicates the optimal number of variables. Roc curve (A, B and C) to evaluate specificity and sensibility of the models (A: UPJO vs transient dilatation, B: UPJO vs Control, C: Transient dilatation vs Control).

**Table S1: a. Epidemiological data for the controls, TD and UPJO patients, b. ANOVA table between control, TD and UPJO group.**

**Table S2: List of 100 metabolites** **identified after NMR analysis including their respective HMDB identifier number.**

**Fig. S1: Superposed of non-normalized average ^1^H NMR spectra of urine samples from control *versus* UPJO (A) UPJO and TD (B).**


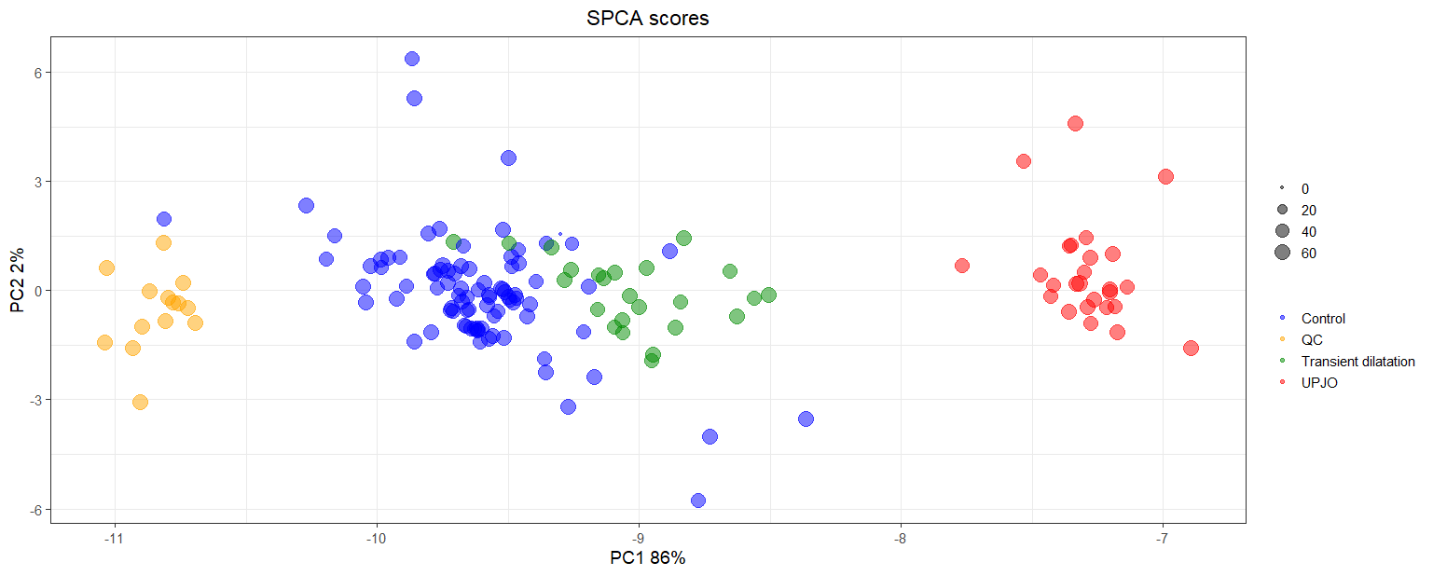


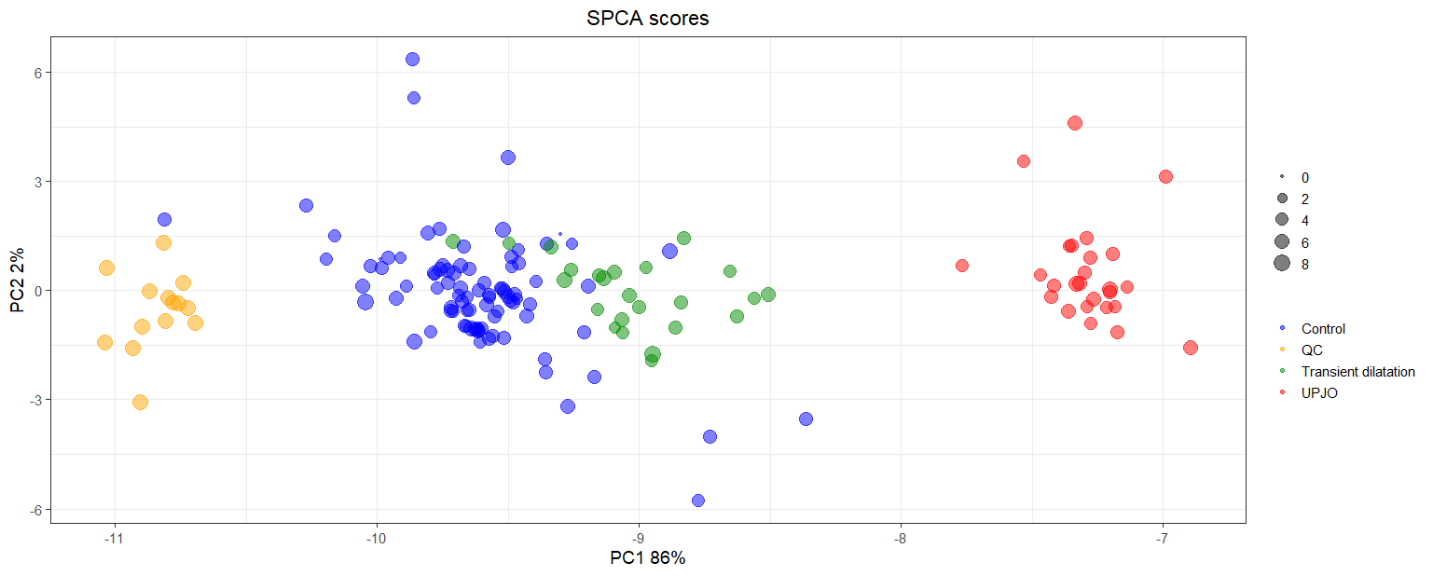


**Fig. S2 : SPCA analysis of metabolites in urine after ^1^H NMR analysis.** SPCA scores with 86 % of the variance explained on the first component and 2 % on the second component; 50 variables are retained on each component; SPCA allows the separation between the different groups; the size of the dots vary according to the height (A) and the weight (B).


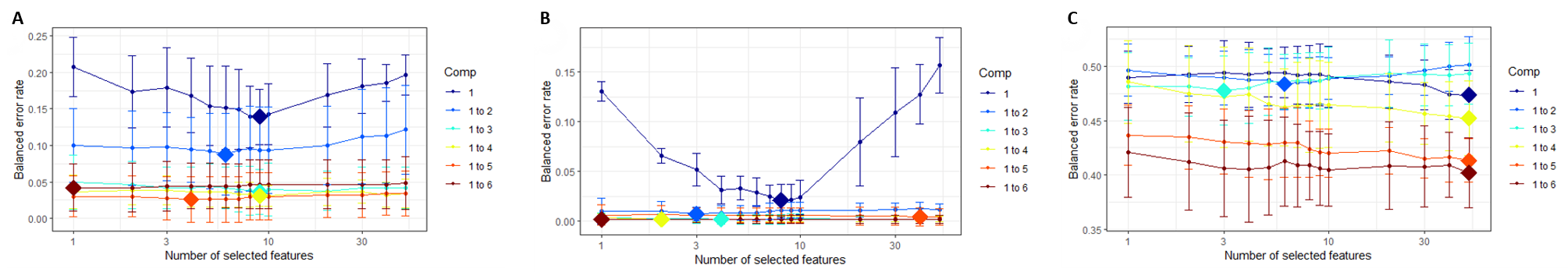


**Fig. S3: Evolution of Error balance rate according to the number of variables selected by components.** A diamond indicates the optimal number of variables. Roc curve (A, B and C) to evaluate specificity and sensibility of the models (A: UPJO vs transient dilatation, B: UPJO vs Control, C: Transient dilatation vs Control)

**Table S1: a. Epidemiological data for the controls, TD and UPJO patients.**

|  |  | Female | Male |
| --- | --- | --- | --- |
| **Control (n=90)** | Age average (days) | 64 | 68.3 |
|  | Age Standard Deviation | 25.4 | 22.7 |
|  | Height average (cm) | 56.2 | 56.3 |
|  | Height Standard Deviation | 3.9 | 4.1 |
|  | Weight average (kg) | 4.9 | 4.9 |
|  | Weight Standard Deviation | 1.3 | 1.3 |
| **Transient Dilatation (n=24)** | Age average (days) | 50 | 56.3 |
|  | Age Standard Deviation | 15 | 20 |
|  | Height average (cm) | 55.4 | 56.7 |
|  | Height Standard Deviation | 3.4 | 4 |
|  | Weight average (kg) | 4.6 | 5.2 |
|  | Weight Standard Deviation | 0.4 | 1.0 |
| **UPJO (n=26)** | Age average (days) | 61.5 | 64.2 |
|  | Age Standard Deviation | 32.4 | 26.4 |
|  | Height average (cm) | 4.9 | 4.9 |
|  | Height Standard Deviation | 1.1 | 0.9 |
|  | Weight average (kg) | 4.9 | 5.1 |
|  | Weight Standard Deviation | 1,9 | 2,1 |

1. **ANOVA table between control, TD and UPJO group**

|  | Age | Height | Weight |
| --- | --- | --- | --- |
| F (Observed Value) | 0,010 | 0,133 | 0,531 |
| F (Critical value) | 3,905 | 3,905 | 3,905 |
| p-value | 0,922 | 0,790 | 0,467 |

**Table S2: List of 100 metabolites** **identified after NMR analysis including their respective HMDB identifier number.**

| 1. 1-Methylhistidine (HMDB00001) | 1. 1-Methylnicotinamide (HMDB00699) |
| --- | --- |
| 1. 1,3-Diaminopropane (HMDB00002) | 1. 1,6-anhydro-β-D-Glucose (HMDB00640) |
| 1. 2-Hydroxyisobutyrate (HMDB00729) | 1. 3-Aminoisobutyrate (HMDB03911) |
| 1. 3-Hydroxyisovalerate (HMDB00754) | 1. 3-Hydroxymethylglutarate (HMDB00355) |
| 1. 4-Hydroxyproline (HMDB00725) | 1. 5-Aminopentanoate (HMDB03355) |
| 1. Acetate(HMDB00042) | 1. Acetoacetate (HMDB00060) |
| 1. Acetone (HMDB01659) | 1. Adipate (HMDB00448) |
| 1. ADP (HMDB01341) | 1. Agmatine (HMDB01432) |
| 1. Allantoin (HMDB00462) | 1. α-ketoisovalerate (HMDB04260) |
| 1. Aminoadipate (HMDB00510) | 1. Ascorbate (HMDB00044) |
| 1. β-alanine (HMDB00056) | 1. Betaine (HMDB00043) |
| 1. Carnosine (HMDB00033) | 1. Choline (HMDB00097) |
| 1. cis-Aconitic acid(HMDB00072) | 1. Citramalate (HMDB00426) |
| 1. Citrate (HMDB00094) | 1. Creatine (HMDB00064) |
| 1. Creatinine (HMDB00562) | 1. Cyclohexanone (HMDB03315) |
| 1. Glucuronic acid (HMDB00127) | 1. Glucose (HMDB00122) |
| 1. Dimethylamine (HMDB00087) | 1. Dimethylglycine (HMDB00092) |
| 1. Dimethyl sulfone (HMDB04983) | 1. Threitol (HMDB04136) |
| 1. Erythritol (HMDB02994) | 1. Ethanolamine (HMDB00149) |
| 1. Formate (HMDB00142) | 1. Fumarate (HMDB00134) |
| 1. Galactitol (HMDB00107) | 1. Galactose (HMDB00143) |
| 1. Glucarate (HMDB00663) | 1. Gluconate (HMDB00625) |
| 1. Glycine (HMDB00123) | 1. Guanidoacetate (HMDB00128) |
| 1. Hippurate (HMDB00714) | 1. Homocarnosine (HMDB00745) |
| 1. Hypoxanthine (HMDB00157) | 1. Isobutyrate (HMDB01873) |
| 1. Isocitrate (HMDB00193) | 1. Acetylcarnitine (HMDB00201) |
| 1. Lactose (HMDB00186) | 1. Alanine (HMDB00161) |
| 1. α-aminobutyrate (HMDB00452) | 1. Arabinose (HMDB00646) |
| 1. Arginine (HMDB00517) | 1. Carnitine (HMDB00062) |
| 1. Fucose (HMDB00174) | 1. Glutamine (HMDB00641) |
| 1. Histidine (HMDB00177) | 1. Lactate (HMDB00190) |
| 1. Lysine (HMDB00182) | 1. Octanoylcarnitine (HMDB00791) |
| 1. Phenylalanine (HMDB00159) | 1. Serine (HMDB00187) |
| 1. Tyrosine (HMDB00158) | 1. Valine (HMDB00883) |
| 1. Mannitol (HMDB00765) | 1. Methylguanidine (HMDB01522) |
| 1. Myoinositol (HMDB00211) | 1. N-Acetylglutamine (HMDB06029) |
| 1. N-Acetyl-L-aspartate (HMDB00812) | 1. N-Acetyl-L-tyrosine (HMDB00866) |
| 1. N-Acetylornithine (HMDB03357) | 1. N-Acetyl-D-glucosamine (HMDB00215) |
| 1. N-Acetylputrescine (HMDB02064) | 1. N,N dimethylaniline (HMDB01020) |
| 1. O-Phosphoethanolamine (HMDB00224) | 1. Ornithine (HMDB00214) |
| 1. Oxoglutarate (HMDB00208) | 1. Pantothenate (HMDB00210) |
| 1. Phenylacetate (HMDB00209) | 1. Phenylacetylglycine (HMDB00821) |
| 1. Pimelate (HMDB00857) | 1. p-Hydroxyphenylacetate (HMDB00020) |
| 1. Propylene glycol (HMDB01881) | 1. Pseudouridine (HMDB00767) |
| 1. Pyroglutamate (HMDB00267) | 1. Pyruvate (HMDB00243) |
| 1. Quinolinate (HMDB00232) | 1. Sorbitol (HMDB00247) |
| 1. Suberate (HMDB00893) | 1. Succinate (HMDB00254) |
| 1. Taurine (HMDB00251) | 1. Threonate (HMDB00943) |
| 1. trans-Aconitate (HMDB00958) | 1. Trimethylamine N-oxide (HMDB00925) |
| 1. Urea (HMDB00294) | 1. Xanthosine (HMDB00299) |
